# Contribution of the EssC ATPase to the assembly of the type 7b secretion system in *Staphylococcus aureus*

**DOI:** 10.1016/j.jbc.2022.102318

**Published:** 2022-07-31

**Authors:** Maksym Bobrovskyy, So Young Oh, Dominique Missiakas

**Affiliations:** 1 Department of Microbiology, University of Chicago, Chicago, Illinois, USA; 2 Howard Taylor Ricketts Laboratory, University of Chicago, Lemont, Illinois, USA

**Keywords:** protein secretion, protein complex, membrane protein, ATPases associated with diverse cellular activities, *Staphylococcus aureus*, Gram-positive bacteria, AAA, ATPase Associated with various cellular Activities, DDM, *n*-dodecyl β-d-maltoside, DUF, domain of unknown function, ESS, ESAT-6 like secretion system, FHA, forkhead-associated domain, T7bSS, type 7b secretion system, TS, Twin-Strep

## Abstract

Secretion systems utilize ATPase activity to facilitate the translocation of proteins into and across membranes. In bacteria, the universally conserved SecA ATPase binds a large repertoire of preproteins and interacts with the SecYEG translocon. In contrast, the type 7b secretion system (T7bSS) of *Staphylococcus aureus* supports the secretion of a restricted subset of proteins. T7bSSs are found in several Firmicutes as gene clusters encoding secreted WXG100 proteins and FtsK/SpoIIIE-like ATPase. In *S. aureus*, this ATPase is called EssC and comprises two cytosolic forkhead-associated domains (FHA_1–2_), two membrane-spanning segments (TM_1–2_), and four cytosolic modules named DUF (domain of unknown function) and ATPases_1-3_ (D1D2D3). However, a detailed understanding of the interactions of EssC in the T7bSS is not clear. Here, we tagged EssC and performed affinity chromatography of detergent-solubilized extracts of wild type and isogenic mutants of *S. aureus*. We found that EssC recruits EsaA, EssA, and EssB in a complex referred to as the ESS (ESAT-6 like secretion system) translocon, and secreted substrates were not required for translocon assembly. Furthermore, deletions of FHA_1_ and DUF rendered EssC unstable, whereas FHA_2_ was required for association with EssB. This interaction was independent of EsaA, but EsaA was required to recruit EssA to the EssC–EssB complex. Finally, we show that assembly of the ESS translocon was impaired upon mutation of D2 structural motifs. Together, our data indicate that the ESS translocon is maintained fully assembled at the plasma membrane and that D2 is fundamental in sustaining the integrity of this complex.

Protein secretion across membranes is facilitated by channel-forming proteins and often powered by ATP or the proton motive force. Dedicated secretion systems control the selective entry of proteins into cognate secretion channels as well as the exit site where substrates are released (1, 2). The final location of secreted substrates and the number of lipid layers crossed during this process account for the diversity and complexity of secretion machinery. For example, in bacteria, preproteins recognized by the SecA ATPase are delivered to the membrane-embedded SecYEG translocon (3). In the absence of additional topogenic or retention sequences, SecA substrates end up in the periplasm of Gram-negative bacteria or the extracellular milieu of Gram-positive bacterial cultures. Although the repertoire of SecA substrates is extensive and highly variable, translocation is highly efficient and preproteins do not accumulate in the cytosol. This is unlike secretion mediated by the highly specialized type 7b secretion system (T7bSS). In *Staphylococcus aureus*, the T7bSS is encoded by the ESS (ESAT-6 like secretion system) gene cluster, which encompasses both transport and secreted proteins (4). T7bSSs are found in several families of Firmicutes, including Bacillaceae, Listeriaceae, Staphylococcaceae, and Clostridiaceae, and are defined by the presence of an ATPase that belongs to the FtsK/SpoIIIE family of proteins and at least one small protein of approximately 100 amino acids with the central Trp-X-Gly motif (WXG100 domain) (5, 6). The association of WXG100 and FtsK/SpoIIIE family of proteins was first described based on a comparative genomics study of Firmicutes and Actinomyces (5) to account for the extracellular location of *Mycobacterium tuberculosis* WXG100 proteins, ESAT-6 and CFP-10 (7). ESAT-6 and CFP-10 are encoded by the adjacent *esxA* and *esxB* genes within the ESX-1 gene cluster, the first of five related clusters ultimately defined as Sec-independent secretion systems in mycobacterial species (8). The remaining genes within ESX clusters of *Actinomyces* and ESS clusters of Firmicutes are not conserved (4, 5, 9, 10, 11). Further ESX clusters have expanded their secretion repertoire to the PE/PPE family of proteins, whereas ESS clusters secrete proteins of the LXG family (12, 13). The two secretion systems are thus referred as T7a and T7b, respectively (8). Most *S. aureus* strains encode two canonical WXG100 proteins, EsxA and EsxB, and two WXG100-like proteins, EsxC and EsxD (14, 15, 16). The current model is that each protein folds into a hairpin of two antiparallel α-helices bent around the WXG motif (when present) and associates as homodimers or heterodimers resulting in tetrahelical bundles (15, 17, 18, 19). Unlike Sec-dependent substrates, WXG100 proteins are not processed during secretion, and folded dimers accumulate in the cytosol. WXG100 proteins are encountered in both pathogenic and environmental species of bacteria. In *S. aureus*, mutants unable to perform T7b secretion are less virulent in the mouse model of bloodstream infection, yet the exact role of T7b during infection remains unknown (4, 20). T7 secretion of larger proteins with an N-terminal WXG100-like domain was first reported in *Bacillus anthracis* (21). Bioinformatic analyses redefined this class of substrates as the LXG family of polymorphic toxins (PFAM: PF04740) (22) and identified a genomic association with immunity factors that were postulated to prevent self-intoxication by cognate polymorphic toxins (22, 23). Multiple copies of seemingly redundant immunity proteins have been proposed to protect from LXG-mediated antagonism between closely related strains (22, 24). Two such proteins, EssD (EsaD) and TspA, were identified in the culture medium of *S. aureus* (25, 26, 27, 28, 29) and have been proposed to mediate intraspecies competition *in vitro* (28, 29). The latter implies translocation of polymorphic toxins across the envelopes of both attacking and prey cells by a mechanism not fully understood (10, 11). Nevertheless, in the attacking cell, EssD (EsaD) interacts tightly with its immunity factor EssI (EsaG) and proposed chaperone EssE (EsaE) (25, 26, 27, 28).

Earlier studies found that EssB, a protein with one transmembrane domain, copurified with three other membrane proteins of the ESS cluster, EsaA, EssA, and EssC (30). Here, we tagged EssC to further characterize the ESS/T7b translocon. We selected EssC because it is conserved with the T7a ATPase EccC, which has been shown to oligomerize and bind WXG100 substrates. Furthermore, soluble domains of T7b EssC have been crystallized from various Firmicutes, including *S. aureus* and *Geobacillus thermodenitrificans*, and can be compared with partial X-ray structures of *Thermomonospora curvata* and *Mycobacterium smegmatis* EccC homologs (31, 32, 33, 34, 35). EssC has also been proposed to promote substrate selection and translocation (36, 37). After generating and purifying several variants of EssC, we report that the N-terminal FHA_2_ (forkhead-associated domain) and C-terminal D2 domains of EssC contribute to the assembly and stability of the ESS translocon. We also find that the translocon assembles in the absence of the secreted substrates encoded within the ESS cluster.

## Results

### Purification and composition of the T7b–ESS membrane complex

**Figure 1:**
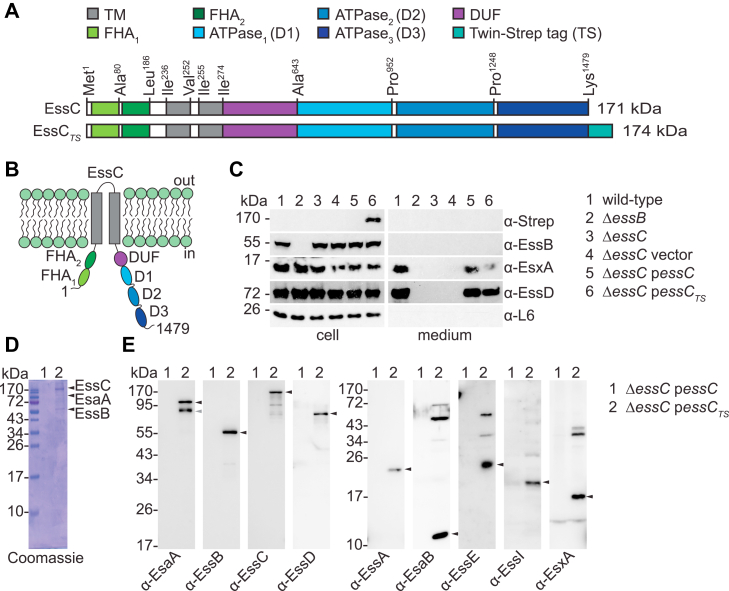
**Purification of the ESS membrane complex of *Staphylococcus aureus* USA300.***A*, depiction of domain organization of recombinant EssC and tagged EssC_TS_ proteins. D: ATPase domain (*shades of blue*); DUF, domain of unknown function (*purple*); FHA, forkhead-associated domain (*light and dark green*); TM, transmembrane domain (*gray*); TS, Twin-Strep tag (*turquoise*). *B*, predicted topology of EssC. Colors for each subdomain are as in *A*. *C*, cultures of *S. aureus* fractionated between cells (cell) and culture medium (*medium*). Proteins in samples were separated by SDS-PAGE and transferred to PVDF membranes for immunoblot analyses, and polyclonal sera are indicated to the *right* of each blot. Number on top of blot identifies the bacterial culture. Ribosomal protein L6 (α-L6) served as a loading and fractionation control. *D* and *E*, DDM extracts were prepared from strains Δ*essC* p*essC* (lane 1, control) and Δ*essC* p*essC*_*TS*_ (lane 2) and purified over Strep-Tactin Sepharose resins. Proteins in eluates were separated by SDS-PAGE, and the gel was stained with Coomassie (*D*) or electrotransferred to PVDF membranes for immunoblot analyses with indicated polyclonal sera (*E*). *Black arrows* indicate immunoreactive species corresponding to the expected size of the proteins of interest. *Gray arrow* indicates an alternative immunoreactive EsaA species. DDM, *n*-dodecyl β-d-maltoside; ESS, ESAT-6 like secretion system; PVDF, polyvinylidene difluoride.

In *S. aureus* strain USA300 FPR3727 (38), herein used as the wildtype strain, the *essC* gene encodes a protein of 1479 amino acids. Two hydrophobic segments, Ile^236^–Val^252^ and Ile^255^–Ile^274^, span the plasma membrane twice, positioning the N and C termini of EssC in the cytosol; only a very small loop faces the exterior of the cell (Fig. 1, *A* and *B*). X-ray crystallography studies revealed that the soluble N-terminal segment folds into two distinct FHA domains comprised of 9 and 12 stacked β-sheets, respectively (31). The cytosolic C-terminal sequence encompasses the domain of unknown function (DUF) followed by three subdomains with characteristic ATPase-like folds. Herein, EssC subdomains are designated FHA_1–2_, TM_1–2_, DUF, and D1D2D3, respectively (Fig. 1, *A* and *B*). D1 and D2 share the greatest amino acid conservation amongst EssC homologs of Firmicutes (Fig. S1*A*). FHA domains are conspicuously missing in most strains of *B. anthracis* (Fig. S1*A*) and are absent in the five EccC proteins (EccC1–5) of *M. tuberculosis* H37Rv (Fig. S1*B*). Overall, the sequences of EccC proteins are clearly distinct from that of *S. aureus* EssC (Fig. S1, *B* and *C*).

To gain insights into the biochemical activity of EssC, we sought to purify the protein from membrane fractions of *S. aureus*. To facilitate purification, the amino-acid sequence WSHPQFEKGGGSGGGSGGSSAWSHPQFEK or Twin-Strep (TS) tag was inserted at the C terminus (Fig. 1*A*). To verify that the tag did not alter the activity of EssC, a secretion assay was performed (4, 14, 20). USA300 lacking *essC* (Δ*essC*) was transformed with plasmids expressing untagged (p*essC*) and tagged *essC* (p*essC*_*TS*_). Cultures of the new strains were spun to separate bacterial cells (cell) and the extracellular medium (medium). Cells were lysed with lysostaphin and proteins in cell and medium compartments, separated by SDS-PAGE, and subjected to Western blot analyses (Fig. 1*C*). The presence of immune reactive species for EsxA and EssD in spent culture media indicated that Ess_TS_ complemented the loss of chromosomally encoded EssC. As controls, secretion was abolished in Δ*essB* and Δ*essC* strains; since the ribosomal protein L6 (L6) sedimented with cellular fractions exclusively, secretion was not because of nonspecific cell lysis (Fig. 1*C*). Next, bacterial cultures were used to extract membrane proteins using *n*-dodecyl β-d-maltoside (DDM), and cleared DDM extracts were applied over Strep-Tactin Sepharose. Bound proteins were eluted with desthiobiotin for LC/MS/MS (Table 1), SDS-PAGE (Fig. 1*D*), and Western blot (Fig. 1*E*) analyses. LC/MS/MS revealed an enrichment of peptides for the conserved ESS proteins, EsaA, EssA, EssB, and EssC in Δ*essC* p*essC*_*TS*_ samples as compared with the Δ*essC* p*essC* control (Table 1). A few peptides also mapped to EsxA, EsaB, and DUF5079 (encoded by *SAUSA300_0295*) (Table 1). Owing to their small sizes, EsxA and EsaB digests yielded fewer peptides. DUF5079 is encoded in ESS clusters, but its function is not known; the gene is often duplicated and could encode an orphan immunity factor. A protein encoded by *SAUSA300_0274* (four genes upstream of *esxA-SAUSA300_0278*) was also identified by LC/MS/MS (Table 1). Subsequent analysis of a strain deleted for *SAUSA300_0274* demonstrated that the gene is dispensable for T7b secretion (Fig. S2). Further investigation into *SAUSA300_0274* was not pursued.

**Table 1:** Identification of proteins copurifying with EssC and EssC_TS_ using LC/MS/MS

Protein	Molecular weight (kDa)	Total number of peptides	
		EssC	EssC_TS_
EssC	170.82	43	653
EssB	51.99	5	108
EsaA	114.75	0	47
SAUSA300_0274	57.89	1	29
EssA	17.38	0	24
EsaB	9.11	0	3
EsxA	11.03	0	2
SAUSA300_0295	15.64	0	1

SDS-PAGE analysis of Strep-Tactin eluates revealed discrete bands that stained with Coomassie brilliant blue for the EssC_TS_ extract but not the untagged EssC control (Fig. 1*D*). Similarly, immunoblotting identified the transmembrane proteins EsaA, EssA, and EssB as well as soluble EsxA and EsaB in EssC_TS_ but not from EssC purification, thereby validating the mass spectrometry results (Fig. 1*E*). Although not identified by LC/MS/MS, the secreted effector EssD, its immunity factor EssI, and putative chaperone EssE were also found in the EssC_TS_ eluate (Fig. 1*E*). Of note, protease inhibitors were used during the extraction procedure, yet multiple immune reactive species were observed for several proteins, in particular EsaA and EssC (*vide infra*). It is not clear whether such processing is the result of nonspecific proteolytic activity or regulated proteolysis integral to the T7bSS.

### FHA_2_ recruits EssB and EssA to the translocon

**Figure 2:**
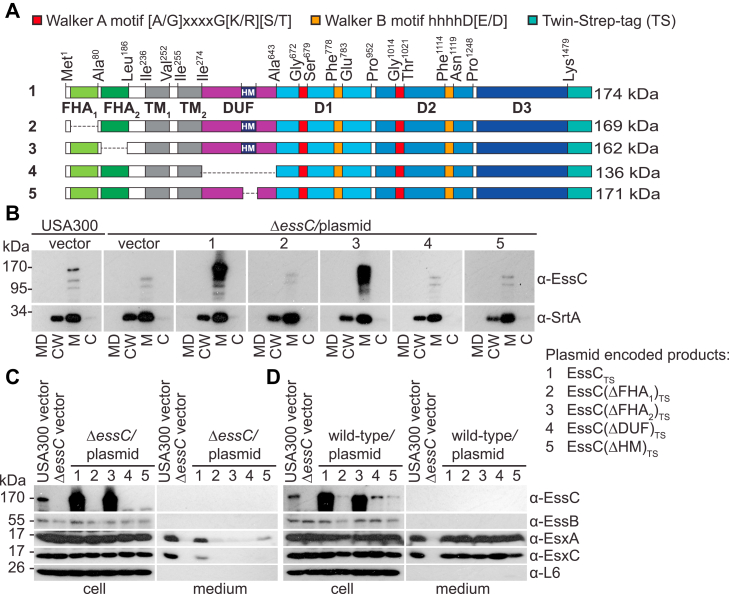
**T7b secretion in mutants lacking FHA domains and DUF.***A*, illustration of EssC_TS_ and its variants EssC(ΔFHA_1_)_TS_, EssC(ΔFHA_2_)_TS_, EssC(ΔDUF)_TS_, and EssC(ΔHM)_TS_. Color code for each domain is as for Figure 1*A*. Walker A and B motifs of D1 and D2 are labeled in *red* and *orange*, respectively. *B*, cultures of *Staphylococcus aureus* strains (as indicated above blot and following numbering code 1–5 to the *right*) were separated into medium (MD), cell wall (CW), membrane (M), and cytosolic (C) fractions and analyzed by immunoblot using rabbit sera: α-EssC and α-SrtA (as a membrane fractionation control). *C* and *D*, cultures of *S. aureus*, Δ*essC* (*C*) or wildtype (USA300, *D*) bearing the vector control or plasmids (1, 2, 3, 4, 5) as indicated to the *right* were separated into medium, and cell fractions and proteins in samples were visualized by immunoblot using rabbit sera as indicated to the *right* of the blots. DUF, domain of unknown function; FHA, forkhead-associated domain.

**Figure 3:**
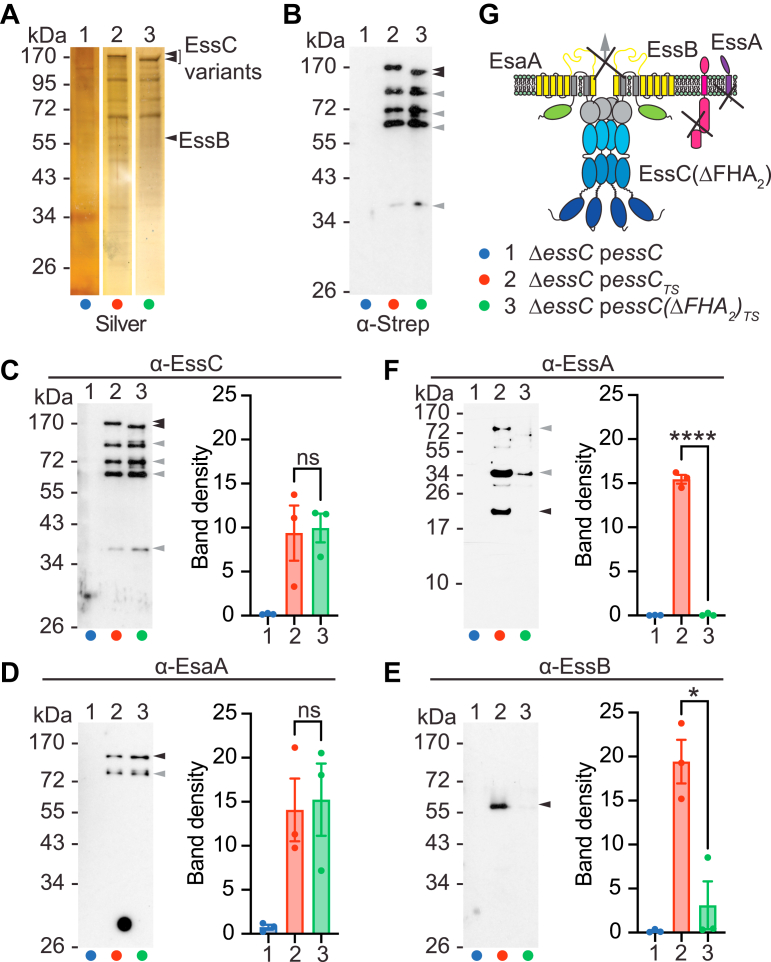
**Assembly of EssC(ΔFHA**_**2**_**)**_**TS**_**in the ESS membrane complex.** DDM extracts were prepared from the indicated strains and purified over Strep-Tactin Sepharose resin. Proteins in eluates were separated by SDS-PAGE, and the gel was stained with silver (*A*) or electrotransferred to PVDF membranes for immunoblot analyses with α-Strep (*B*) or with indicated polyclonal sera, α-EssC (*C*), α-EsaA (*D*), α-EssB (*E*), α-EssA (*F*). *Black arrows* indicate species for which band densities from three replicate experiments were quantified using ImageJ (54) and analyzed with an unpaired parametric *t* test. *p* > 0.1234 (ns), 0.0332 (∗), 0.0021 (∗∗), 0.0002 (∗∗∗), and <0.0001 (∗∗∗∗). *Gray arrows* indicate alternative immunoreactive species. *G*, illustration depicting the loss of interactions between EssC(ΔFHA_2_) and EssB-EssA but not with EsaA. DDM, *n*-dodecyl β-d-maltoside; ESS, ESAT-6 like secretion system; FHA, forkhead-associated domain; ns, not significant; PVDF, polyvinylidene difluoride.

The two FHA domains of EssC share 23.21% identity and are altogether missing in the EccC1–5 of *M. tuberculosis* as well as in *B. anthracis* strains that retained EssC but lack EsaA, EssB, EssA, and EsaB (Fig. S1, *C* and *D*) (21). Plasmid constructs were generated to produce tagged EssC lacking each one of the FHA domains yielding EssC(ΔFHA_1_)_TS_ and EssC(ΔFHA_2_)_TS_, respectively (Fig. 2*A*). This analysis was extended to DUF and a small predicted hydrophobic segment (HM) within DUF yielding EssC(ΔDUF)_TS_ and EssC(ΔHM)_TS_, respectively (Fig. 2*A*). The four new plasmids were transformed in strain Δ*essC* for immunoblotting analyses (Fig. 2, *B* and *C*). Constructs lacking FHA_1_, DUF, or HM did not yield any product that could be detected with the anti-EssC polyclonal serum (Fig. 2, *B* and *C*). Only EssC(ΔFHA_2_)_TS_ accumulated to the same extent as EssC_TS_ (Fig. 2, *B* and *C*). Subcellular fractionation of bacteria demonstrated that EssC(ΔFHA_2_)_TS_ retained its membrane location (Fig. 2*B*) but did not support the secretion of EsxA and EsxC (Fig. 2*C*). Immunoblotting for the membrane protein Sortase A (SrtA), ribosomal protein L6, and EssB served as controls (Fig. 2, *B* and *C*). The four plasmids were also transformed in the wildtype strain. Only EssC_TS_ and EssC(ΔFHA_2_)_TS_ accumulated to a similar extent (Fig. 2*D*). Surprisingly, overproduced EssC(ΔFHA_2_) did not exert a dominant-negative function on EsxA and EsxC secretion (Fig. 2*D*). Together, the results suggest that the deletions of FHA_1_, DUF, and HM render EssC unstable. This is in agreement with a report suggesting that deletion of both FHA domains renders EssC unstable (31). This instability can be attributed solely to the loss of FHA_1_. Stable EssC(ΔFHA_2_) did not interfere with the ESS translocon, either because it was not recruited to the translocon or because incorporation of these defective subunits was tolerated (Fig. 2*D*). To look into the role of FHA_2_ further, DDM solubilized extracts were prepared from cultures of strain Δ*essC* harboring plasmid-encoded EssC(ΔFHA_2_)_TS_ as well as EssC_TS_ and EssC for controls. All extracts were purified over Strep-Tactin Sepharose, and proteins in eluates were separated by SDS-PAGE for silver staining (Fig. 3*A*) and immunoblot analyses (Fig. 3, *B*–*F*). As expected, binding to the beads was only observed for tagged proteins (Fig. 3, *A*–*F*; lane 1). Both bound EssC_TS_ and EssC(ΔFHA_2_)_TS_ recruited EsaA (Fig. 3, *B*–*D*; lanes 2–3). However, in the absence of FHA_2_, EssC no longer interacted with EssB and EssA (Fig. 3, *E* and *F*; lane 3). Because this partial complex was still embedded in the membrane (Fig. 3*G*) and cells remained viable, we conclude that incomplete EssC–EsaA translocons are not leaky.

**Figure 4:**
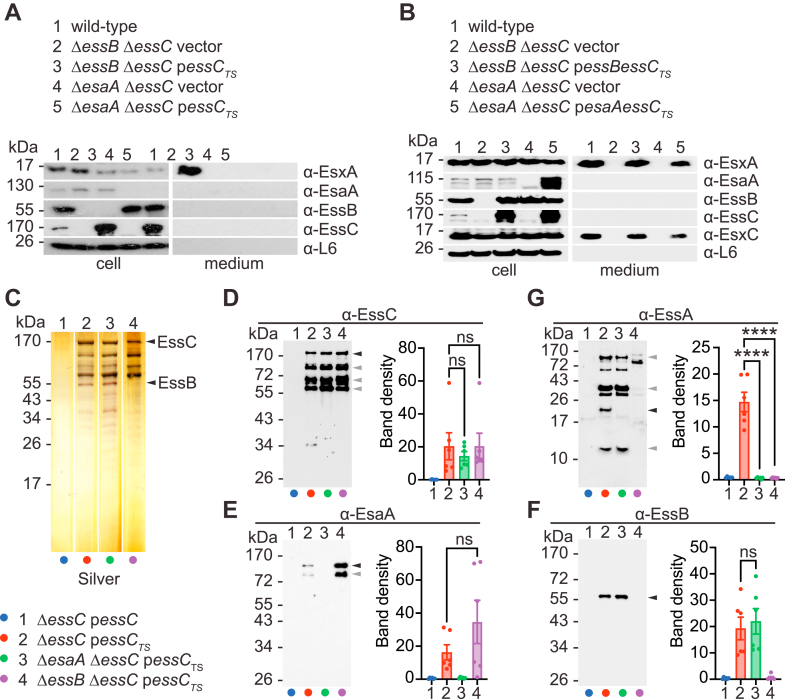
**ESS complex assembly in the absence of EsaA or EssB.***A* and *B*, cultures of *Staphylococcus aureus* strains (as indicated on *panel*) were centrifuged to separate proteins in cell and medium fractions. Proteins in samples were separated by SDS-PAGE and transferred to PVDF membranes for immunoblot analyses, and polyclonal sera are indicated to the *right* of each blot. *C*–*G*, DDM extracts were prepared from strains 1 to 4 with color code listed in *B* and purified over Strep-Tactin Sepharose resin. Proteins in eluates were separated by SDS-PAGE, and the gel was stained with silver (*C*) or electrotransferred to PVDF membranes for immunoblot analyses with indicated polyclonal sera α-EssC (*D*), α-EsaA (*E*), α-EssB (*F*), and α-EssA (*G*). *Black arrows* indicate species for which band densities from three replicate experiments were quantified using ImageJ (54) and analyzed with an unpaired parametric *t* test. *p* > 0.1234 (ns), 0.0332 (∗), 0.0021 (∗∗), 0.0002 (∗∗∗), and <0.0001 (∗∗∗∗). *Gray arrows* indicate alternative immunoreactive species. DDM, *n*-dodecyl β-d-maltoside; ESS, ESAT-6 like secretion system; ns, not significant; PVDF, polyvinylidene difluoride.

To further explore the formation of partial ESS complexes, the p*essC*_*TS*_ plasmid or vector control was introduced in the double mutant strains *ΔesaAΔessC* and Δ*essB*Δ*essC*. Cultures of the four new strains were separated into cell and medium fractions and validated by Western blot using sera against EsxA, EsaA, EssB, EssC, as well as secreted EssH and cytosolic ribosomal protein L6 as controls (Fig. 4*A*). As expected, secretion of EsxA was abrogated in all these strains, but no other differences were noted. For instance, deletion of *esaA* did not impact the production of EssB and vice versa (Fig. 4*A*). As additional controls, the double mutant strains *ΔesaAΔessC* and Δ*essB*Δ*essC* were complemented with a plasmid expressing either genes *esaA* and *essC* (p*esaAessC*_*TS*_) or genes *essB* and *essC* (p*essBessC*_*TS*_). In both cases, EsxA and EsxC secretion was restored (Fig. 4*B*). Next, DDM-soluble fractions of strains bearing p*essC*_*TS*_ or vector were purified over Strep-Tactin. Silver staining (Fig. 4*C*) and Western blotting (Fig. 4*D*) revealed that comparable amounts of EssC_TS_ were produced in the single *ΔessC* and the double mutant strains *ΔesaAΔessC* and Δ*essB*Δ*essC* (Fig. 4, *C* and *D*). As noted earlier, although protease inhibitors were added during sample preparation, four immune reactive species were observed for EssC regardless of the strain background (Fig. 4, *C* and *D*). Immunoblot analyses also revealed that EsaA copurified with EssC_TS_ in the absence of EssB (Fig. 4*E*) and likewise EssB copurified with EssC_TS_ in the absence of EsaA (Fig. 4*F*). However, EssA no longer associated with EssC_TS_ in the absence of either EsaA or EssB (Fig. 4*G*). Together, the data suggest that EssC can associate with EsaA and EssB independently, but EssA interaction requires both associations.

### D2 is required for the assembly of the ESS translocon

**Figure 5:**
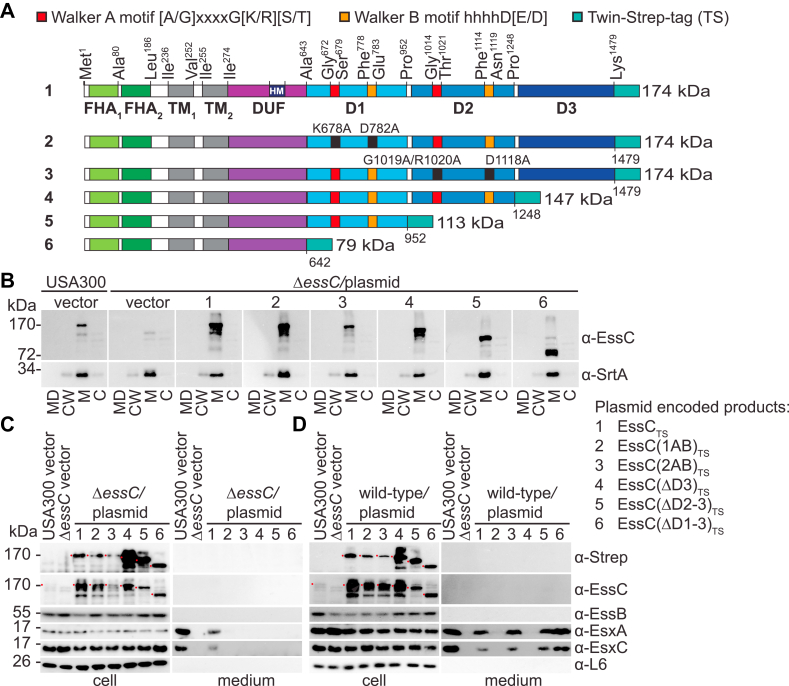
**T7b secretion in mutants bearing altered ATPase domains.***A*, domain organization of EssC_TS_ and its variants. Color code for each domain is as indicated for Figure 1*A*. *B*, cultures of *Staphylococcus aureus* strains (as indicated in the figure) were separated into medium (MD), cell wall (CW), membrane (M), and cytosolic (C) fractions and analyzed by immunoblot using rabbit sera: α-EssC and α-SrtA (as a membrane fractionation control). *C* and *D*, cultures of *S. aureus*, Δ*essC* (*C*) or wildtype (USA300, *D*) bearing the vector control or plasmids as indicated to the *right* were separated into cell and medium fractions, and proteins in samples visualized by immunoblot using rabbit sera as indicated to the *right* of the blots.

**Figure 6:**
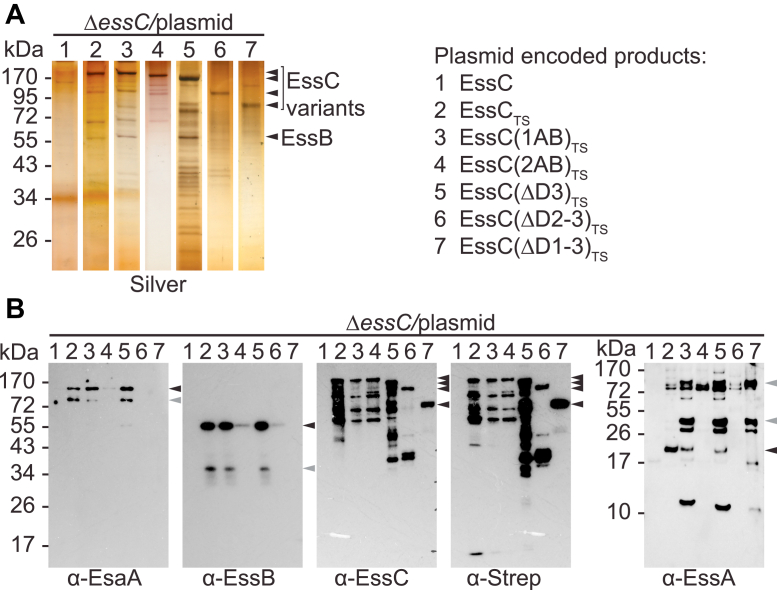
**Purification of ESS complexes with deletions or substitutions in various ATPase domains.***A*, DDM extracts were prepared from the indicated strains (1, 2, 3, 4, 5, 6, 7) and purified over Strep-Tactin Sepharose resin. Proteins in eluates were separated by SDS-PAGE, and the gel was stained with silver or (*B*) electrotransferred to PVDF membranes for immunoblot analyses with indicated polyclonal sera. *Black arrows* indicate immunoreactive species corresponding to the expected size of the proteins of interest. *Gray arrows* indicate alternative immunoreactive species. DDM, *n*-dodecyl β-d-maltoside; ESS, ESAT-6 like secretion system; PVDF, polyvinylidene difluoride.

The three FtsK/SpoIIIE-like domains, D1D2D3, complete the C terminus of EssC (Figs. 1*B* and 5*A*). Only D1 and D2 contain canonical ATP-binding catalytic Walker A ([A/G]xxxxG[K/R][S/T]) and Walker B (hhhhD[E/D]) motifs. To evaluate the contribution of these domains for T7b secretion, plasmids encoding C-terminally TS-tagged *essC* variants lacking D3 [EssC(ΔD3)_TS_], D2 and D3 [EssC(ΔD2–3)_TS_], or D1 through D3 [EssC(ΔD1–3)_TS_] were generated (Fig. 5*A*). Similarly, two variants with substitutions in Walker A and B motifs were also constructed: EssC(1AB)_TS_ with substitutions K678A (Walker A) and D782A (Walker B), and EssC(2AB)_TS_ with substitutions G1019A/R1020A (Walker A) and D1118A (Walker B) (Fig. 5*A*). Subcellular fractionation of bacteria demonstrated that the new substitutions did not affect the membrane localization of EssC (Fig. 5*B*). None of the truncated variants or point mutants restored the secretion of EsxA and EsxC in strain *ΔessC* (Fig. 5*C*). However, when expressed in the wildtype strain, EssC lacking D3 [EssC(ΔD3)] inhibited the activity of the translocon as documented by the loss of EsxA and EsxC secretion (Fig. 5*D*). This inhibitory activity was not observed with subsequent deletions of D2 [EssC(ΔD2–3)] and D1 [EssC(ΔD1–3)] (Fig. 5*D*). Substitutions in Walker A and Walker B motifs of D1, but not D2, also interfered with the activity of the translocon as plasmid expression of *essC(1AB)*_*TS*_ resulted in a dominant-negative phenotype over chromosomally expressed *essC* for the secretion of EsxA and EsxC (Fig. 5*D*). We speculate that D2 activity is critical for complex formation since variants EssC(1AB) and EssC(ΔD3) (with intact D2) interfered with the wildtype translocon and inhibited T7b secretion, whereas EssC(2AB), EssC(ΔD2–3), and EssC(ΔD1–3) did not. Next, DDM extracts of the *ΔessC* strain carrying plasmid-encoded EssC_TS_ variants were purified over Strep-Tactin Sepharose and eluates examined by silver staining of SDS-PAGE (Fig. 6*A*) or immunoblot (Fig. 6*B*). EssC(ΔD1–3) lacking all three ATPase domains was quite stable as compared with all other variants, including full-length EssC. EssC(ΔD1–3) failed to copurify with EsaA, EssA, and EssB. EssC(ΔD2–3) and EssC(2AB) behaved similarly (Fig. 6*B*). The absence of D3 or substitutions in the Walker A/B motifs of D1 did not affect the association of EssC with EsaA and EssB (Fig. 6*B*). In summary, all ATPase domains are required for substrate secretion. However, D2 is also critical for the assembly of the ESS membrane complex.

### EssC association with EsaA, EssA, and EssB does not require ESS-encoded substrates

**Figure 7:**
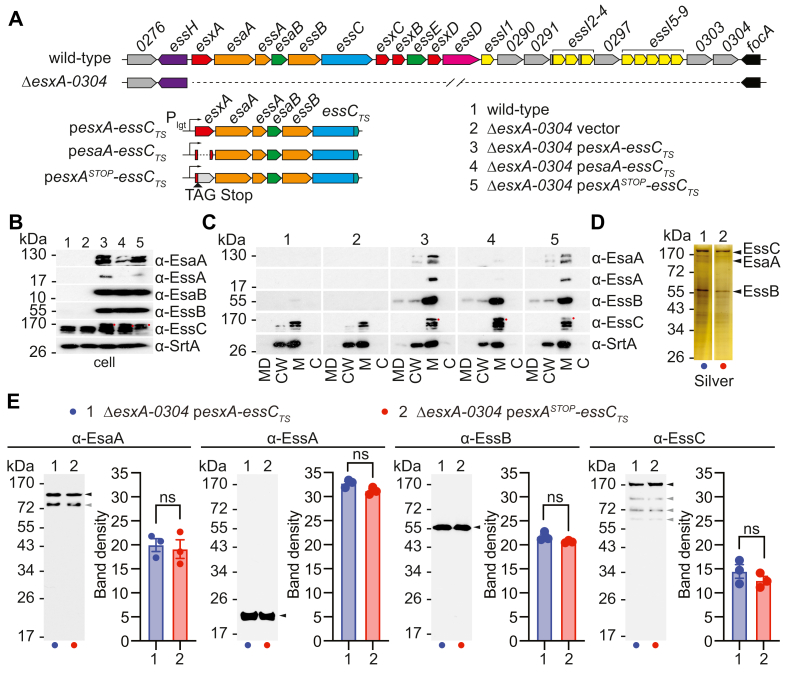
**Purification of the ESS membrane complex in the absence of secreted substrates.***A*, illustration of strains and plasmids used in experiments shown in the figure. *B*, Western blot analyses of cell extracts from strains (1, 2, 3, 4, 5) depicted in *A*. *C*, cultures of *Staphylococcus aureus* strains (1, 2, 3, 4, 5) were separated into medium (MD), cell wall (CW), membrane (M), and cytosolic (C) fractions and analyzed by immunoblot using rabbit sera as indicated to the *right* of the blots. *D*, DDM extracts were prepared from two strains as indicated *above* the panel and purified over Strep-Tactin Sepharose resin. Proteins in eluates were separated by SDS-PAGE, and the gel was stained with silver or (*E*) electrotransferred to PVDF membranes for immunoblot analyses with indicated polyclonal sera. *Black arrows* indicate species for which band densities from three replicate experiments were quantified using ImageJ (54) and analyzed with an unpaired parametric *t* test. *p* > 0.1234 (ns). *Gray arrows* indicate alternative immunoreactive species. DDM, *n*-dodecyl β-d-maltoside; ESS, ESAT-6 like secretion system; ns, not significant; PVDF, polyvinylidene difluoride.

Our results indicated that D2 contributes to the assembly of the ESS translocon. In an independent study, Mietrach *et al.* (37) used fluorophore-labeled D2D3 and D3 of USA300 EssC (as used in this study) to report an interaction between D2 and purified EsxB. Our LC/MS/MS analysis of purified EssC_TS_ identified the secreted substrate EsxA as a possible interacting partner of the ESS translocon (Table 1). Thus, we wondered if ESS-encoded WXG100 proteins participate in the assembly of the translocon *via* D2 interactions. To test this possibility, a mutant lacking gene *esxA* through *SAUSA300_0304* (Δ*esxA*-*0304*) and thus also lacking *esxB*, *esxC*, and *esxD*, was constructed along with two plasmids, p*esxA-essC*_*TS*_ and p*esaA-essC*_*TS*_ (Fig. 7*A*). In this experiment, only one WXG100 protein is provided on plasmid p*esxA-essC*_*TS*_ (Fig. 7*A*). Although expression and production of recombinant genes were driven by the constitutive *lgt* promoter and a strong Shine–Dalgarno sequence (39), production of EsaA and EssA was not observed when plasmid p*esaA-essC*_*TS*_ was introduced in strain Δ*esxA*-*0304* (Fig. 7*B*). When the clone included gene *esxA* (plasmid p*esxA-essC*_*TS*_), production of EsaA and EssA was restored (Fig. 7*B*). When *esxA* was modified to introduce a stop codon at Glu 10 of EsxA (plasmid p*esxA*^*STOP*^*-essC*_*TS*_), production of EsaA and EssA in strain Δ*esxA-0304* was also restored (Fig. 7, *A* and *B*). Subcellular fractionation experiments confirmed that plasmid-borne EsaA, EssA, EssB, and EssC retained their membrane localization in strain Δ*esxA-0304* (Fig. 7*C*). Next, TS-tagged proteins were purified from cultures of strain Δ*esxA-0304* carrying plasmid p*esxA-essC*_*TS*_ or p*esxA*^*STOP*^*-essC*_*TS*_ (Fig. 7*D*). EsaA, EssA, and EssB copurified with EssC_TS_ (Fig. 7, *D* and *E*). The data show that ESS-encoded WXG and LXG substrates are not required for the assembly of the T7b membrane complex. Nonetheless, in *S. aureus*, USA300 *esxA* bears sequence information that is important for the optimal expression of downstream genes *esaA* and *essA*. The lost information cannot be compensated by providing a strong constitutive promoter and Shine–Dalgarno sequence.

## Discussion

The T7a and T7b secretion systems borrow an ATPase from the FtsK/SpoIIIE family of the FtsK–HerA superfamily of P-loop ATPases within the larger AAA+ superfamily (ATPases Associated with various cellular Activities) (40, 41, 42). Characteristics of the AAA+ superfamily include the presence of a phosphate-binding loop (P-loop) or Walker A motif ([A/G]xxxxG[K/R][S/T]) forming the nucleotide-binding pocket and Walker B motif (hhhhD[E/D]) and arginine finger (R-finger) for ATP hydrolysis (41, 43). ATPase activity requires interaction between adjacent subunits forming a ring with Walker residues contributed by the *cis* subunit and R-finger contributed by the adjacent *trans* subunit (43). Thus, oligomerization completes the ATP-binding pocket, with most AAA+ proteins forming ring-shaped hexamers for ATP hydrolysis–driven translocation of protein or DNA substrates through a central channel. Protein translocation by AAA+ ATPases results in unfolding, whereas DNA translocation leads to unwinding during replication or packaging of viral genomes (40, 41, 42, 44). For example, FtsK proteins assemble into membrane-tethered ring-shaped hexamers at bacterial septa to translocate naked double-stranded DNA into daughter cells. FtsK activity can be described as treadmilling along the spiral staircase DNA substrate, a process essentially like cytomotive protein filaments, but in a closed circular arrangement (45, 46).

**Figure 8:**
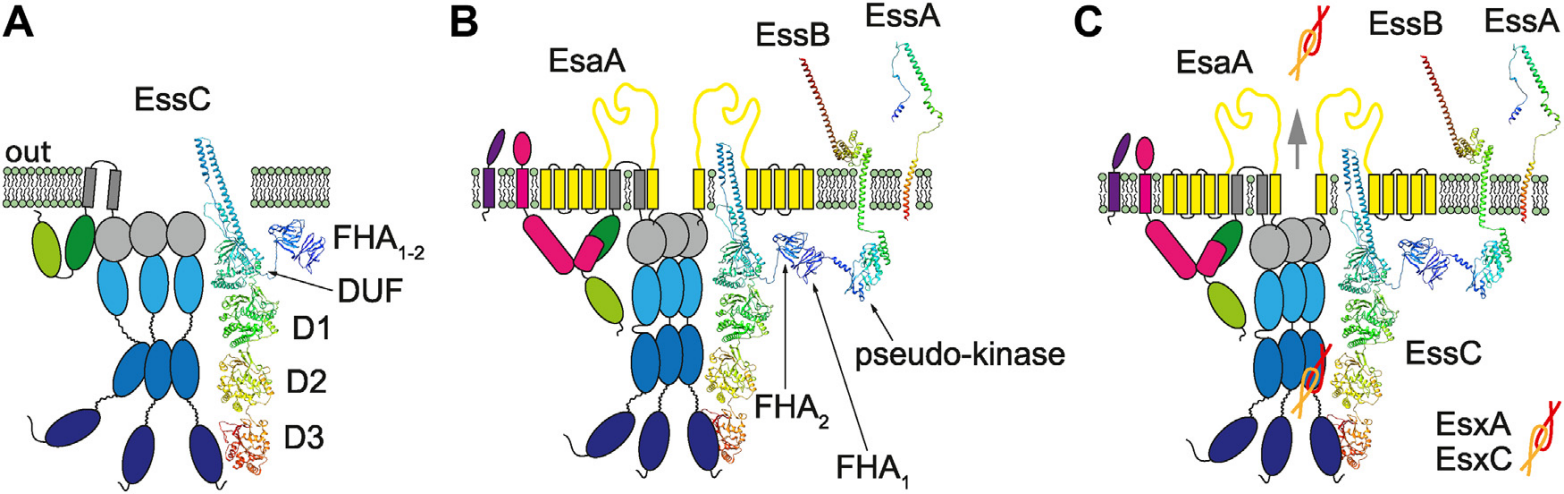
**Modeling the ESS membrane complex.***A*, EssC multimerization requires a functional D2 domain (*shade of blue*). *B*, EssC interacts *via* its FHA_2_ domain (*shade of green*) with the cytosolic domain of EssB (*pink*) encompassing a pseudokinase fold. The exact association between EssC, EsaA, and EssA remains to be determined. Assembly between EssC, EsaA, EssA, and EssB does not require ESS-encoded WXG100 substrates but is lost when the Walker A and B motifs of D2 are mutated. *C*, substrates interact with the ESS membrane complex, which directs them for secretion. Protein structures for EssC, EssB, and EssA were predicted using AlphaFold (55). ESAT-6 like secretion system; FHA, forkhead-associated domain.

As far as we know, EssC proteins of Firmicutes translocate proteins, not DNA. Like the related EccC (T7a) of *Actinomyces* and unlike cell division FtsK proteins, T7b EssC proteins contain multiple structural ATP-binding domains. By tagging EssC and using a combination of mass spectroscopic and immunoblot analyses, we determined that EssC copurifies with EsxA, EsaA, EssA, EsaB, and EssB following detergent extraction of membrane proteins from wildtype *S. aureus*. By generating a strain unable to produce EsxA and downstream WXG/LXG products, we ruled out the possibility that these secreted substrates are required for the assembly of this DDM extractable complex. Using similar approaches, we asked how each subdomain may contribute to secretion and complex assembly. We generated EssC variants lacking either FHA_1_, FHA_2_, DUF or with increasing C-terminal deletions (ΔD3, ΔD2–3, and ΔD1–3) or with mutations in Walker A/B motifs of D1 or D2. None of these variants supported protein secretion as reported by others (31, 37). Removal of FHA_1_ or DUF, including the small HM segment within DUF, failed to produce EssC altogether. We do not have an explanation for these observations. We found that EssC associates with EssB and EssA *via* FHA_2_, a unique structural feature of T7bSS (Fig. 8, *A* and *B*). FHA domains are commonly found in eukaryotes where they often participate in signal transduction processes and interact with phosphorylated ligands to modulate cell cycle, chromosome segregation, and protein degradation (47). Only a few FHA-containing bacterial proteins have been characterized thus far. In the plant pathogen *Agrobacterium tumefaciens*, the protein known as Fha binds phosphothreonine–modified TssL, an inner-membrane core component of the type 6 secretion system, affecting its assembly and secretion (48). Here, we found that EssC lacking FHA_2_ does not associate with EssB and EssA but retains interactions with EsaA (Fig. 8*B*). While we are not yet aware of phosphothreonine or phosphoserine modification in T7b proteins, the cytosolic domain of EssB has been shown to adopt a pseudokinase fold and mediate dimerization of EssB (49). Studies in *Bacillus subtilis* have shown that the isolated pseudokinase domain of YukC (EssB homolog) and FHA_1–2_ of YukB (EssC homolog) interact (50). Thus, in *S. aureus*, the pseudokinase and FHA_2_ domains might also be sufficient to establish an interaction between EssB and EssC to prompt EssA recruitment. Indeed, EssA did not associate with the translocon in the absence of EssB.

When focusing on the C terminus of EssC, we found that D3 is dispensable for complex assembly since the truncated variant, EssC(ΔD3), pulled down the EsaA–EssA–EssB complex. EssC(ΔD3) also exerted a dominant-negative phenotype for secretion likely because it was able to form mixed EssC(ΔD3)–EssC–EsaA–EssA–EssB complexes. When we examined other mutants, EssC(ΔD2–3), EssC(ΔD1–3), or substitutions in the Walker A/B motifs of D1 or D2, we found that the dominant-negative phenotype correlated with the ability to pull down EsaA–EssA–EssB. Our results suggest that the Walker A/B motifs of D2 are critical for bringing or keeping the ESS membrane complex (EssC–EsaA–EssA–EssB) together. While not described here, we failed to measure ATP hydrolysis using recombinant full-length EssC or smaller fragments regardless of incubation with WXG100 substrates. Zolner *et al.* (31) solved the structure of D2D3 of *G. thermodenitrificans Gt*EssC (*Gt*D2D3) and found intact ATP in the nucleotide-binding site of *Gt*D2 but not *Gt*D3, which points to weak intrinsic ATPase activity. Mietrach *et al.* (37) reported the crystal structures of *S. aureus* D3 (same *Sa*D3 as the one used in this study) and by comparing structures of *Gt*EssC and *T. curvata* EccC (*Tc*EccC) proposed that the ATP-binding sites for *Sa*D3 and *Gt*D3 may be structurally occluded as compared with T7a D3 domains (35, 37). D3 domains in all three species (*Gt*, *Sa*, and *Tc*) carry a degenerated Walker B motif with no distinguishable R finger. D3 of *S. aureus* could be mutated to either increase or reduce T7b secretion once again suggesting a contribution for ATP binding or hydrolysis (37). In *T. curvata*, the soluble *Tc*D1D2D3 domain was shown to form dimers and higher-order oligomers in the presence of *Tc*EsxB, whereas *Tc*EsxA resulted in oligomer dissociation *in vitro* (35). A model was proposed whereby substrates with a C-terminal secretion motif (*e.g.*, EsxB of *M. tuberculosis* or *T. curvata*) dock into the exposed nucleotide-binding site of *Tc*D3, triggering a conformational change transduced along the EccC monomer (35). In this model, ATPases are inhibited by subunit interactions along the EccC monomer, and the substrate relieves inhibition as it is moved upward. This model is different from the proposed mechanism of canonical FtsK (AAA+ ATPases) whereby conformational changes are transduced *via* the R-finger when subunits are brought together in the hexameric ring.

Secretion motifs in staphylococcal T7b substrates have been difficult to pinpoint (14), yet Warner *et al.* (51) noted that highly variable sequences were associated with D3 and immediate downstream genes encoding strain-specific WXG and LXG effectors. Swapping of D3 between strains supported the notion of EssC selectivity toward nonconserved effectors but not toward conserved EsxA (36). Thus, D3 is likely to play a role in substrate recruitment. Yet, isolated D2 has also been shown to interact with EsxB and EsxC of *S. aureus* (37). We surmise that oligomerization of *Sa*EssC may first be imposed by its 12 membrane-spanning segments. This is modeled on the proposed structures of EccC3 and EccC5 revealed by recent cryo-EM images of mycobacterial ESX-3 and ESX-5 complexes (34, 52). In *S. aureus*, at least two EsaA molecules (each containing seven transmembrane segments) could be wrapped around the EssC hexamer (Fig. 8*C*). Interestingly, DUF may also adopt a Rossman fold as demonstrated for EccC3 of *M. smegmatis* ESX-3 (34). Thus, the C terminus of *Sa*EssC may contain four domains with similar structures (D0–D1–D2–D3), with D1 and D2 carrying canonical Walker A and B motifs and R-fingers. We propose that R734 and R1078 in D1 and D2 of *Sa*EssC allow for the formation of two stacked rings under the transmembrane and D0 segments, introducing rigidity into EssC hexamers and perhaps providing for opening and closing into a conduit that extends across the plasma membrane (Fig. 8*C*). The Walker A/B motifs of D2 are essential for oligomerization and assembly of the ESS complex, and D0 is required for protein stability. How other proteins in this complex, ATP and ultimately secreted substrates, influence conformational changes and gating of this complex remain to be elucidated.

## Experimental procedures

### Media and growth conditions

*S. aureus* strains were cultured in tryptic soy broth or tryptic soy agar at 37 °C, unless otherwise stated, and media were supplemented with 10 μg/ml chloramphenicol for plasmid selection and 0.2% heat-inactivated horse serum (Gibco/Life Technologies) to trigger secretion.

### Bacterial strains and plasmids

Relevant strains and plasmids used in this study are listed in Table S1 and primers in Table S2. *S. aureus* USA300 LAC∗, a clone of the epidemic community–acquired methicillin-resistant *S. aureus* strain (38) was used as the wildtype strain. Plasmid DNA was passaged in *S. aureus* RN4220 before transformation into USA300 strains. Mutations in strain USA300 LAC∗ were introduced by pKOR1-mediated allelic replacement (53). Plasmids pKOR1-Δ*essC*, pKOR1-Δ*essB*Δ*essC*, and pKOR1-Δ*esxA-0304* were constructed by PCR amplifying upstream and downstream DNA sequences flanking the desired region to be deleted. The following primer pairs were used for amplification: pKOR1-*ΔessC* primers 112F/112R and 128F/128R; pKOR1-Δ*essB*Δ*essC* primers 106F/106R and 107F/107R; and pKOR1-Δ*esxA-0304* primers 97F/97R-XbaI and 98F-XbaI/98R. DNA fragments for pKOR1-Δ*essC* and pKOR1-Δ*essB*Δ*essC* were stitched by PCR and for pKOR1-Δ*esxA-0304* by restriction digestion using XbaI and ligation. Stitched DNA fragments were cloned into pKOR1 using BP Clonase Kit (Invitrogen). Mutants Δ*essC*, *ΔessB*Δ*essC*, and Δ*esxA-0304* were constructed as described (53). Mutant Δ*esaA*Δ*essC* was generated by transforming Δ*esaA* strain with pKOR1-Δ*essC*, and Δ*essC* was introduced by allelic replacement. Plasmids p*essC* and p*essC*_*TS*_ were constructed by PCR amplification of *essC* gene from *S. aureus* USA300 LAC∗ genomic DNA using primer pairs 557/572 or 557/563, respectively. PCR products were cloned into the pWWW412 vector at the XhoI/BamHI sites (39). Plasmids p*essC(ΔD3)*_*TS*_, p*essC(ΔD2-3)*_*TS*_, and p*essC(ΔD1-3)*_*TS*_ were constructed using p*essC*_*TS*_ as a template for PCR amplification of intermediate linear products using primers pairs 661/662, 660/662, and 659/662, respectively. Linear PCR products were digested with BamHI (as well as DpnI) and religated to generate circular plasmids encoding truncated *essC*_*TS*_ variants. Plasmid p*essC(1AB)*_*TS*_ was constructed by PCR amplification and Gibson assembly (NEB) of four DNA fragments from p*essC*_*TS*_ template using four primer pairs 663/670, 665/666, 667/668, and 669/664. Plasmid p*essC(2AB)*_*TS*_ was constructed by QuikChange PCR mutagenesis using p*essC*_*TS*_ as template. Primer pair 156Fmut/156Rmut was used to introduce G1019A,R1020A in Walker A and 157Fmut/157Rmut for D1118A in Walker B. Plasmids p*essC(ΔFHA*_*1*_*)*_*TS*_, p*essC(ΔFHA*_*2*_*)*_*TS*_, p*essC(ΔDUF)*_*TS*_, and p*essC(ΔHM)*_*TS*_ were constructed using p*essC*_*TS*_ as a template for PCR amplification of intermediate linear product using overlapping 5′-phosphorylated primer pairs 173F-P/173R-P, 174F-P/174R-P, 175F-P/175R-P, and 176F-P/176R-P, respectively. Linear PCR products were religated to generate circular plasmids encoding truncated *essC*_*TS*_ variants. Plasmids p*esxA-essC*_*TS*_, p*esaA-essC*_*TS*_, and p*esxA*^*STOP*^*-essC*_*TS*_ were constructed by PCR amplification of *esxA-essC*, *esaA-essC*, and *esxA*^*STOP*^*-essC* fragments from *S. aureus* USA300 LAC∗ genomic DNA using primer pairs 121F/121R, 135F/121R, and 203F/121R, respectively. XhoI/BglII-digested DNA products and XhoI/BamHI-digested p*essC*_*TS*_ plasmid were ligated using DNA Ligase (NEB) to generate p*esxA-essC*_*TS*_, p*esaA-essC*_*TS*_, and p*esxA*^*STOP*^-*essC*_*TS*_. To generate plasmids p*esaAessC*_*TS*_ and p*essBessC*_*TS*_ (these two plasmids encode two genes each), plasmid p*esxA*^*STOP*^*-essC*_*TS*_ was used as a template, and linear products were obtained by PCR using overlapping 5′-phosphorylated primer pairs 230F-P/230R-P and 231F-P/231R-P. The primer pairs were designed to eliminate the intervening *essA-esaB-essB* and *esaA-essA-esaB* encoding sequences, respectively.

### Fractionation of bacterial cultures

For protein secretion and subcellular fractionation assays, *S. aureus* culture aliquots were spun at 10,000*g* for 10 min to separate proteins in the supernatant (medium) and cells. To assess for protein secretion, cells in bacterial pellets were washed and lysed using lysostaphin (10 μg/ml for 1 h at 37 °C) yielding lysed cell extracts. Proteins in these suspensions (medium or cell) were precipitated by addition of 12% final concentration trichloroacetic acid, washed in cold acetone, and dried. For subcellular localization of proteins, washed cells were suspended in 50 mM Tris–HCl, pH 7.5, 0.5 M sucrose, and 10 mM MgCl_2_ buffer prior to lysostaphin treatment. Samples were spun at 15,000*g* for 10 min, the supernatants (cell wall fraction) were transferred to a new tube, and protoplasts were lysed by repeated freeze thawing (three cycles). The extracts were spun at 100,000*g* for 40 min at 4 °C to separate the supernatants containing cytosolic proteins (cytoplasmic fraction). Pellets containing membrane proteins were suspended in PBS buffer (membrane fraction). Proteins in all fractions were precipitated with trichloroacetic acid, washed with methanol, and dried. All precipitates were solubilized in 100 μl of 0.5 M Tris–HCl (pH 8.0), 4% SDS, and heated at 90 °C for 10 min.

### Purification of the ESS membrane complex

Bacterial cultures were grown to an absorbance of ∼3.0 at 600 nm at 37 °C. Cells were collected by centrifugation and suspended in buffer A (20 mM Tris [pH 8.0], 300 mM NaCl, 10% [v/v] glycerol, SIGMA*FAST* Protease Inhibitors [SIGMA]). Cells were lysed using bead beating, and debris was removed by centrifugation. Soluble and insoluble fractions were separated by centrifugation at 100,000*g* for 1 h. Pellet fractions containing membrane proteins were suspended in buffer A with 0.25% DDM and incubated for 1 h at 4 °C. Insoluble material was removed by centrifugation at 100,000*g* for 30 min, and cleared supernatants were loaded onto 1 ml bed of Strep-Tactin Sepharose resin (IBA). The resin was washed with 10 ml buffer A and incubated for 18 h with 10 ml of buffer A containing 0.5 g/ml Amphipol A8-35 (Anatrace) to replace DDM. The resin was washed with an additional 10 ml of buffer A containing 0.5 g/ml Amphipol A8-35 prior to elution of proteins with buffer A containing 0.5 g/ml Amphipol A8-35 and 5 mM d-desthiobiotin (IBA). Aliquots of eluted fractions were mixed with an equal volume of sample buffer and separated on 12 or 15% SDS-PAGE and stained with Coomassie and silver or transferred to polyvinylidene difluoride membrane for immunoblot analyses.

### Western blot

Proteins separated by SDS-PAGE were transferred to polyvinylidene difluoride membrane. The membranes were blocked for 1 h at room temperature in 10 ml of blocking buffer containing 0.5% fat-free milk prior to the addition of primary polyclonal antibodies (at a dilution of 1:2000). Membranes were incubated for an additional hour, washed three times for 10 min in 50 mM Tris–HCl (pH 7.5), 150 mM NaCl, and 0.1% Tween-20, and incubated with 1:10,000 anti-rabbit or antimouse horseradish peroxidase–conjugated secondary antibody (Cell Signaling Technology) for 1 h. Membranes were washed again in 50 mM Tris–HCl (pH 7.5), 150 mM NaCl, and 0.1% Tween-20, and immunoreactive products were revealed by chemiluminescent detection using SuperSignal West Pico Chemiluminescent Substrate (Thermo Fisher Scientific). Blots were developed on Amersham Hyperfilm ECL (GE Healthcare Life Sciences), and band densities were quantified using ImageJ (National Institutes of Health) (54) software. Graphing and statistical analysis were performed using Prism 9 software (GraphPad Software, Inc).

## Data availability

All data are contained within the article.

## Supporting information

This article contains supporting information (20, 25, 38, 53, 56, 57).

## Conflict of interest

The authors declare that they have no conflicts of interest with the contents of this article.
