# Supplementary material for: Contribution of the EssC ATPase to the assembly of the type 7b secretion system in Staphylococcus aureus
Source: J Biol Chem. 2022 Jul 31;298(9):102318. doi: 10.1016/j.jbc.2022.102318 (PMC9436818; doi:10.1016/j.jbc.2022.102318)
Supplement: Supporting Information [file mmc1.pdf]

**Supporting Information**

# Contribution of the EssC ATPase to the assembly of the type 7b secretion system in *Staphylococcus aureus*

Maksym Bobrovskyy[1], So Young Oh[2], Dominique Missiakas[1,2*]

There are 2 supporting tables, 2 figures with legends, and references list

**Supporting tables**

**Table S1. Strains and plasmids used in this study.**

| Strain or plasmid | Description | References |
|---|---|---|
| **Strains** | | |
| DH5α | *E. coli fhuA2 lacΔU169 phoA glnV44 Φ80′ lacZΔM15 gyrA96 recA1 relA1 endA1 thi-1 hsdR17* | Lab collection |
| USA300 LAC* | *S. aureus* USA300 LAC lacking pUSA03 | (1,2) |
| RN4220 | restriction deficient, cloning intermediate | (3) |
| Δ*essB* | *S. aureus* USA300 LAC* Δ*essB* | (4) |
| Δ*essC* | *S. aureus* USA300 LAC* Δ*essC* | This study |
| Δ*essB* Δ*essC* | *S. aureus* USA300 LAC* Δ*essBC* | This study |
| Δ*esaA* Δ*essC* | *S. aureus* USA300 LAC* Δ*esaA*, Δ*essC* | This study |
| Δ*esxA-0304* | *S. aureus* USA300 LAC Δ*esxA-SAUSA300_0304* | This study |
| **Plasmids** | | |
| pOS1 | *E. coli/S. aureus* shuttle vector | (5) |
| pKOR1 | *E. coli/S. aureus* shuttle vector for allelic replacement in *S. aureus* | (6) |
| pWWW412 | pOS1 derivative herein referred as vector | Laboratory collection |
| p*essC* | $P_{lgt}$-*essC* | This study |
| p*essC*$_{TS}$ | $P_{lgt}$-*essC*$_{TS}$ | This study |
| p*essC(1AB)*$_{TS}$ | $P_{lgt}$-*essC(1AB)*$_{TS}$ | This study |
| p*essC(2AB)*$_{TS}$ | $P_{lgt}$-*essC(2AB)*$_{TS}$ | This study |
| p*essC(ΔD3)*$_{TS}$ | $P_{lgt}$-*essC(ΔD3)*$_{TS}$ | This study |
| p*essC(ΔD2-3)*$_{TS}$ | $P_{lgt}$-*essC(ΔD2-3)*$_{TS}$ | This study |
| p*essC(ΔD1-3)*$_{TS}$ | $P_{lgt}$-*essC(ΔD1-3)*$_{TS}$ | This study |
| p*essC(ΔFHA1)*$_{TS}$ | $P_{lgt}$-*essC(ΔFHA1)*$_{TS}$ | This study |
| p*essC(ΔFHA2)*$_{TS}$ | $P_{lgt}$-*essC(ΔFHA2)*$_{TS}$ | This study |
| p*essC(ΔDUF)*$_{TS}$ | $P_{lgt}$-*essC(ΔDUF)*$_{TS}$ | This study |
| p*essC(ΔHM)*$_{TS}$ | $P_{lgt}$-*essC(ΔHM)*$_{TS}$ | This study |
| p*essBessC*$_{TS}$ | $P_{lgt}$-*essBessC*$_{TS}$ | This study |
| p*esaAessC*$_{TS}$ | $P_{lgt}$-*esxA*$^{STOP}$*esaAessC*$_{TS}$ | This study |
| p*esxA-essC*$_{TS}$ | $P_{lgt}$-*esxAesaAessAesaBessBessC*$_{TS}$ | This study |
| p*esxA*$^{STOP}$*-essC*$_{TS}$ | $P_{lgt}$-*esxA*$^{STOP}$*esaAessAesaBessBessC*$_{TS}$ | This study |
| p*esaA-essC*$_{TS}$ | $P_{lgt}$-*esaAessAesaBessBessC*$_{TS}$ | This study |

**Table S2. Primers used in this study.**

| Primer | Sequence (5'⟶3') |
|---|---|
| 97F | GGGGACAAGTTTGTACAAAAAAGCAGGCTTTAATGGATGTAATTATAT GATGAAACTTCTGAAGCAGAGATGG |
| 97R-XbaI | GGGGTCTAGATCTTAATCATTGCCATAACTAGAAACCTCCTG |
| 98F-XbaI | GGGGTCTAGAAGCGACTTATCATAAACATCGTATATTGAAATTTCAAG ATTTC |
| 98R | GGGGACCACTTTGTACAAGAAAGCTGGGTGGTAAATAACGTAGTCGA AACGATAAGAACAAAAGATATATTAC |
| 106F | GGGGACAAGTTTGTACAAAAAAGCAGGCTGCTGGCTATAGAGCGAAA TTCATCTTAATG |
| 106R | GTTATGATTTTTAACCATCTATTTTTCCTCCTATAGTAACTTC |
| 107F | GAAGTTACTATAGGAGGAAAAATAGATGGTTAAAAATCATAACAGAT GGTTTAAATAGCAATGAATTAAATAGGAGGGAG |
| 107R | GGGGACCACTTTGTACAAGAAAGCTGGGTCATAGCTAAATGGTGCAA AATACATATCATTAATTCG |
| 112F | CAGGAGGTTTCTAGTTATGGCAATGATTAAGAGATGGTTTAAATAGCA ATGAATTAAATAGGAGGGAGG |
| 112R | GGGGACCACTTTGTACAAGAAAGCTGGGTCAATAAAGGATATGATTG TACAATCGACCACC |
| 121F | GGGGCTCGAGATGGCAATGATTAAGATGAGTCCAGAGG |
| 121R | GGGGAGATCTTTTAAACCATCTAATCTTTTGATAAGCTTGGTTTGC |
| 128F | GGGGACAAGTTTGTACAAAAAAGCAGGCTGAAGAAGTAAATCGTACA CGCTACACATTTG |
| 128R-XbaI | GGGGTCTAGACAATTTATGCATTGTCTTTGCCTCAGTCC |
| 135F | GGGGCTCGAGATGAAAAAGAAAAATTGGATTTATGCATTAATTGTCA C |
| 156Fmut | GTTAATCGGAAGTCCAGGATATGcagcAACAACGTTCTTAC |
| 156Rmut | CGAAAATAATGTTGTGTAAGAACGTTGTTgctgCATATCCTGG |
| 157Fmut | CAATTCCGCATGTCTTTATTCTTATTGcgAACTTTGACGC |
| 157Rmut | GAAAGGTGAATCTTTTACTGCGTCAAAGTTcgCAATAAGAATAAAG |
| 173F-P | /5Phos/TCATTTGCGTATCCTTCAATTCAAGATACGATG |
| 173R-P | /5Phos/GACTTCGCCTAACGATTTCAACGTAATATCTGC |
| 174F-P | /5Phos/TTAACACAAGAGATGCCACATGCACAGG |
| 174R-P | /5Phos/TGCATAATCAGCTTCTGTATAAAGCTGTAATGTAATGTCATC |
| 175F-P | /5Phos/GAGATGTATAACGTGAAAGAAGTAGATCAGCTTGATGTG |
| 175R-P | /5Phos/GTAATCTTTCTCTCGTTTTTCAACATCTTTGTTATAC |
| 176F-P | /5Phos/GAATATGTAAACCAAGATTTATCAGAATATGGTATTTCATTAAT CTTTG |
| 176R-P | /5Phos/TTGCTCATTACTTCTGCTGCGTTCACGCAC |
| 203F | GGGGCTCGAGATGGCAATGATTAAGATGAGTCCAGAG |

| | |
|---|---|
| 230F-P | /5Phos/GCTGAAAGAAAAAAATAGTATAGGACTGAGGCAAAGAC |
| 230R-P | /5Phos/GCTATTCATCAACATTAGATTAATCTCTCTTTCTTAAAGTG |
| 231F-P | /5Phos/ATGGTTAAAAATCATAACCCTAAAAATGAAATGCAAGATATG |
| 231R-P | /5Phos/CTCGAGCATATGTTCACCTCAATTGTATTTATCCC |
| 557 | TTTCTCGAGATGCATAAATTGATTATAAAATATAAC |
| 563 | TTAAGATCTTATTTTTCAAATTGTGGATGTGACCATGCTGAAGAACCT CCACTACCTCCACCACTTCCACCACCTTTTTCAAATTGTGGATGTGACC ATGCGGATCCTTTAAACCATCTAATCTTTTGATAAG |
| 572 | TTTGGATCCTATTTAAACCATCTAATCTTTTGATAAG |
| 659 | AAAAGGATCCCATCGTTTTGTATGTTTCGTTTTG |
| 660 | AAAAGGATCCTGGACGCTTAACTTCTTCGATTTC |
| 661 | AAAAGGATCCAGGAATATCATTTGGTGTTTCACC |
| 662 | CTTATCAAAAGATTAGATGGTTTAAAGGATCC |
| 663 | GCTCGAGATGCATAAATTGATTATAAAATATAACAAACAATTG |
| 664 | CAATTTATGCATCTCGAGCATATGTTCACCTC |
| 665 | GGGCATCTGAGATTATCCAATCATACATTTTATCTTTAG |
| 666 | GGATAATCTCAGATGCCCCTGAACCAGTGGTACCAGC |
| 667 | GCAGAGTTTGCCGAATTAAAATCAGAACAACC |
| 668 | TTCGGCAAACTCTGCGGAAATAATGAATAAATGTG |
| 669 | GGTCACATCCACAATTTGAAAAAGGTGGTGG |
| 670 | CAAATTGTGGATGTGACCATGCGGATCCTTTAAAC |

## Supporting figures

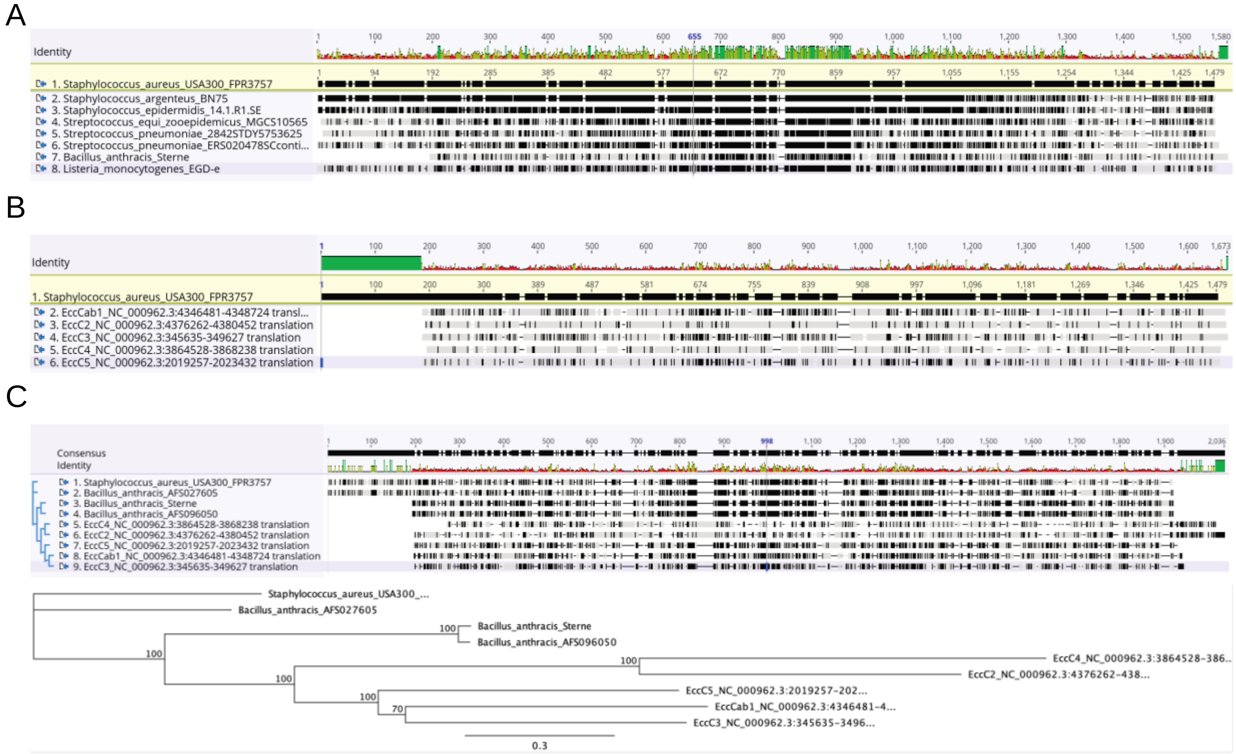


D

```
FHA1 -----------------------------MHKLIIKYNKQLKMLNLRDGKTYTISEDERADITL-----------KSLGEVIHLEQNNQGTWQANHT---------SINKVLVRKGDLDDIT LQLYTEA    1-80
FHA2 DYASFAYPSIQDTMTIGPNAYDDMVIQSLMNAIIIKDFQSI-----QESQYVRIVHDKNTDVYINYELQEQLTNKAYIGDHI----YVEGIWLEVQADGLNVLSQNTVASSLIR-------- -------    81-185
                                  *: :***  :.:     ::.:   * .*:.:*: :           :*: *      :* *    ::         :: . *:*
```

**Figure S1. Comparison of EssC homologues. A)** Amino acid sequence alignment of EssC orthologues from *Staphylococcus aureus* USA300 (1), *Staphylococcus argenteus* BN75 (2), *Staphylococcus epidermidis* 14.1.R1 (3), *Streptococcus equi zooepidemicus* MGCS10565 (4), *Streptococcus pneumoniae* 2842STDY5753625 (5), *S. pneumoniae* ERS020478SC (6), *Bacillus anthracis* Sterne (7) and *Listeria monocytogenes* EGD-e. **B)** Amino acid sequence alignment of EssC from *S. aureus* USA300 (1) with EccC1-5 from *Mycobacterium tuberculosis* H37Rv (2-6). **C)** Amino acid sequence alignment of EssC orthologs from *S. aureus* USA300 (1), *B. anthracis* AFS027605 (2), *B. anthracis* Sterne (3) and *B. anthracis* AFS096050 (4) with EccC1-5 of *M. tuberculosis* H37Rv (5-9). Below, is the neighbor-joining consensus tree based on the sequence alignment above. Residues with 100% identity are marked black, >80% similar are dark grey, 60-80% similar are light grey and <60% similar is near white. **D)** Alignment of EssC domains $FHA_1$ and $FHA_2$ of *S. aureus* USA300. Identical residues are denoted (*), conserved (:) and semi-conserved (.).

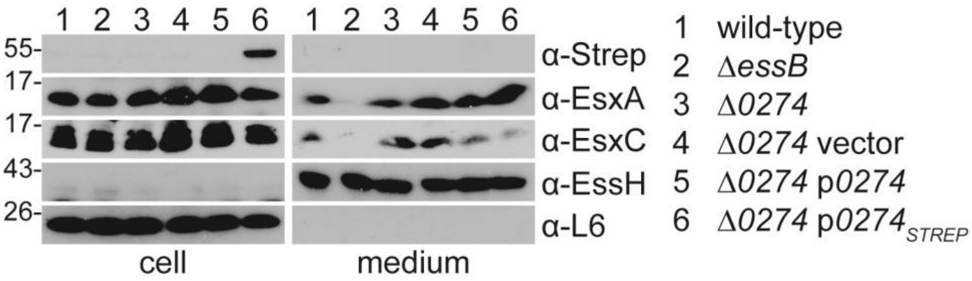


**Figure S2. T7b-dependent secretion in the Δ*0274* mutant strain.** Cultures of *S. aureus* USA300 (wild-type), Δ*essB*, Δ*0274*, Δ*0274* vector, Δ*0274* p*0274* and Δ*0274* p*0274*$_{STREP}$ strains were grown to $A_{600}$ 3.0 and proteins in the extracellular medium (medium) fractionated from staphylococci in the sediment (cell) by centrifugation. Protein extracts from each fraction were evaluated by western blot using rabbit polyclonal antibodies α-EsxA, α-EsxC, α-EssH, α-L6 (ribosomal protein L6) as control, and mouse monoclonal antibodies α-Strep (Strep tag).

## References cited


1. Burts, M. L., DeDent, A. C., and Missiakas, D. M. (2008) EsaC substrate for the ESAT-6 secretion pathway and its role in persistent infections of Staphylococcus aureus. *Mol Microbiol* **69**, 736-746
2. Diep, B. A., Gill, S. R., Chang, R. F., Phan, T. H., Chen, J. H., Davidson, M. G., Lin, F., Lin, J., Carleton, H. A., Mongodin, E. F., Sensabaugh, G. F., and Perdreau-Remington, F. (2006) Complete genome sequence of USA300, an epidemic clone of community-acquired meticillin-resistant *Staphylococcus aureus*. *Lancet* **367**, 731-739
3. Kreiswirth, B. N., Lofdahl, S., Betley, M. J., O'Reilly, M., Schlievert, P. M., Bergdoll, M. S., and Novick, R. P. (1983) The toxic shock syndrome exotoxin structural gene is not detectably transmitted by a prophage. *Nature* **305**, 709-712
4. Anderson, M., Chen, Y. H., Butler, E. K., and Missiakas, D. M. (2011) EsaD, a secretion factor for the Ess pathway in Staphylococcus aureus. *J Bacteriol* **193**, 1583-1589
5. Schneewind, O., Model, P., and Fischetti, V. A. (1992) Sorting of protein A to the staphylococcal cell wall. *Cell* **70**, 267-281
6. Bae, T., and Schneewind, O. (2006) Allelic replacement in Staphylococcus aureus with inducible counter-selection. *Plasmid* **55**, 58-63
